# Draft Genome Sequence of Parageobacillus thermoglucosidasius Strain TG4, a Hydrogenogenic Carboxydotrophic Bacterium Isolated from a Marine Sediment

**DOI:** 10.1128/MRA.01666-18

**Published:** 2019-01-31

**Authors:** Masao Inoue, Ayumi Tanimura, Yusuke Ogami, Taiki Hino, Suguru Okunishi, Hiroto Maeda, Takashi Yoshida, Yoshihiko Sako

**Affiliations:** a Graduate School of Agriculture, Kyoto University, Sakyo-ku, Kyoto, Japan; b Faculty of Fisheries, Kagoshima University, Kagoshima, Japan; Georgia Institute of Technology

## Abstract

Parageobacillus thermoglucosidasius possesses biotechnological potential for fuel generation. Here, we report the draft genome sequence of P. thermoglucosidasius strain TG4, which was first isolated from a marine sediment. The genome sequence provides insight into the plasmid diversity and carbon monoxide-dependent hydrogen production capacity of P. thermoglucosidasius.

## Abstract (precis)

Parageobacillus thermoglucosidasius possesses biotechnological potential for fuel generation. Here, we report the draft genome sequence of P. thermoglucosidasius strain TG4, which was first isolated from a marine sediment.

## ANNOUNCEMENT

Parageobacillus thermoglucosidasius is a Gram-positive thermophilic facultatively anaerobic spore-forming bacterium. This species has biotechnological potential for fuel generation from plant biomass through fermentation (1–3). Recently, P. thermoglucosidasius was also identified as a hydrogenogenic carboxydotroph, suggesting its further potential for energetics (4). The genome sequences of nine strains, including DSM 2542^T^, have already been published (5–9). P. thermoglucosidasius is present in various environments, such as soils, hot springs, and milk plants (3, 7–9). Here, we report the draft genome sequence of P. thermoglucosidasius strain TG4, first isolated from a marine sediment.

The sediment was collected from the Aira Caldera in Kagoshima Bay, Japan (31°38′9″N, 130°46′53″E, 83-m depth). Strain TG4 was enriched at 65°C under 100 % CO gas in B medium (10) modified to contain 1.8% NaCl, 0.07% KCl, 0.03% NH_4_Cl, and 0.39% MgCl_2_ · 6H_2_O. Single-colony isolation was performed aerobically using NBRC 802 agar medium. Cells were grown in B medium supplemented with 0.2% sodium pyruvate under 100 % CO gas.

Genomic DNA was extracted using the DNeasy blood and tissue kit (Qiagen, Hilden, Germany). A DNA library was prepared using the Nextera mate pair library preparation kit (Illumina, San Diego, CA). Sequencing was performed on the Illumina MiSeq instrument with MiSeq reagent kit v.3 (600 cycles), which generated 6,822,150 paired-end reads. Quality trimming and adapter removal were performed using Trimmomatic v.0.3.6 (11). Mate pair reads were selected and junction adapters were trimmed using NxTrim v.0.4.1 (12). *De novo* genome assembly was performed with SPAdes v.3.13.0 (13) using the filtered 3,879,002 mate pair reads. The assembled scaffolds were quality controlled using the Burrows-Wheeler Aligner (BWA) v.0.7.17 (14), SAMtools v.0.1.19 (15), and NxRepair v.0.13 (16). Annotation was performed with the DFAST server v.1.0.2 (17). Genomic comparison was performed using the OrthoANIu tool (18) and BLASTn (19).

The draft genome was assembled into 12 scaffolds, including one circular plasmid, with an *N*_50_ value of 3,846,774 bp, an average coverage of 288.74×, a total length of 3,948,523 bp, and an average G+C content of 43.34%. The numbers of predicted protein-coding genes, rRNAs, and tRNAs were 3,906, 25, and 90, respectively. The orthologous average nucleotide identity with DSM 2542^T^ was 98.9%.

A circular plasmid (1,586 bp) exhibited homology (76% query coverage with 85% identity) to plasmid pGTG5 (1,540 bp) from *Geobacillus* sp. strain 1121 (20). We also found two plasmid-like scaffolds (60,265 and 34,404 bp) that exhibited homology (30% and 99% query coverages with 97% and 99% identities, respectively) to plasmid pNCI001 (83,935 bp) from P. thermoglucosidasius NCIMB 11955 (5). Moreover, we identified a gene cluster of carbon monoxide dehydrogenase/hydrogen-evolving hydrogenase complex, including 15 genes exhibiting ≥99% amino acid identity with those of DSM 2542^T^ (4), which would enable carbon monoxide-dependent hydrogen production.

### Data availability.

The draft genome sequences were deposited in DDBJ/ENA/GenBank under the accession numbers BHZK01000001 to BHZK01000011 (for the scaffolds) and AP019364 (for the circular plasmid). The raw reads were deposited in SRA/DRA/ERA under the accession number DRA007789.
